# Image quality comparisons of coil setups in 3T MRI for brain and head and neck radiotherapy simulations

**DOI:** 10.1002/acm2.13794

**Published:** 2022-10-26

**Authors:** Evangelia Kaza, Jeffrey P. Guenette, Christian V. Guthier, Steven Hatch, Alexander Marques, Lisa Singer, Jonathan D. Schoenfeld

**Affiliations:** ^1^ Radiation Oncology, Brigham and Women's Hospital Dana‐Farber Cancer Institute, Harvard Medical School Boston Massachusetts USA; ^2^ Division of Neuroradiology, Brigham and Women's Hospital Dana‐Farber Cancer Institute, Harvard Medical School Boston Massachusetts USA; ^3^ Radiation Oncology University of California San Francisco California USA

**Keywords:** brain, head and neck, MR coils, MR simulation, radiotherapy treatment planning

## Abstract

**Purpose:**

MRI is increasingly used for brain and head and neck radiotherapy treatment planning due to its superior soft tissue contrast. Flexible array coils can be arranged to encompass treatment immobilization devices, which do not fit in diagnostic head/neck coils. Selecting a flexible coil arrangement to replace a diagnostic coil should rely on image quality characteristics and patient comfort. We compared image quality obtained with a custom UltraFlexLarge18 (UFL18) coil setup against a commercial FlexLarge4 (FL4) coil arrangement, relative to a diagnostic Head/Neck20 (HN20) coil at 3T.

**Methods:**

The large American College of Radiology (ACR) MRI phantom was scanned monthly in the UFL18, FL4, and HN20 coil setup over 2 years, using the ACR series and three clinical sequences. High‐contrast spatial resolution (HCSR), image intensity uniformity (IIU), percent‐signal ghosting (PSG), low‐contrast object detectability (LCOD), signal‐to‐noise ratio (SNR), and geometric accuracy were calculated according to ACR recommendations for each series and coil arrangement. Five healthy volunteers were scanned with the clinical sequences in all three coil setups. SNR, contrast‐to‐noise ratio (CNR) and artifact size were extracted from regions‐of‐interest along the head for each sequence and coil setup. For both experiments, ratios of image quality parameters obtained with UFL18 or FL4 over those from HN20 were formed for each coil setup, grouping the ACR and clinical sequences.

**Results:**

Wilcoxon rank‐sum tests revealed significantly higher (*p* < 0.001) LCOD, IIU and SNR, and lower PSG ratios with UFL18 than FL4 on the phantom for the clinical sequences, with opposite PSG and SNR trends for the ACR series. Similar statistical tests on volunteer data corroborated that SNR ratios with UFL18 (0.58 ± 0.19) were significantly higher (*p* < 0.001) than with FL4 (0.51 ± 0.18) relative to HN20.

**Conclusions:**

The custom UFL18 coil setup was selected for clinical application in MR simulations due to the superior image quality demonstrated on a phantom and volunteers for clinical sequences and increased volunteer comfort.

## 1 INTRODUCTION

Magnetic resonance imaging (MRI) is increasingly used for radiotherapy treatment planning of brain or head and neck cancers due to its variety of image weighting mechanisms and superior soft tissue contrast compared to computed tomography (CT).^1^, ^2^ Advances in magnetic resonance (MR) image acquisition and processing allow the production of synthetic CT images useful for dose calculations, bearing the potential for MR‐only planning.^3^ MRI implementation for treatment planning purposes (MR simulation) necessitates image acquisition in treatment position, with high spatial resolution and geometric fidelity.^4^ In brain and head and neck radiotherapy, motion reduction and accurate tumor localization for imaging and treatment is achieved by fixing the patient's head and possibly neck and shoulders in individually molded thermoplastic masks. These masks are usually fixed on underlying devices, which do not fit in the receiving coils designed for diagnostic head and neck MRI.

As interest in MR scanner implementation for radiotherapy simulation recently increased, vendors currently provide dedicated equipment options to facilitate treatment planning. These include flat tabletops, attachable boards bearing thermoplastic masks, moldable cushions, and positioning aids. Phased array coils, which can be bent to accommodate the body part of interest, can replace rigid diagnostic coils for signal reception. Multiple such flexible coils can be combined to closely surround immobilization devices. A commercial solution for brain and head and neck MR simulations on Siemens scanners relies on image acquisition by two FlexLarge4 (FL4) coils surrounding the head mask, attached side‐by‐side inside a holder placed under the flat tabletop. We propose a new custom solution by using two larger UltraFlexLarge18 (UFL18) coils instead, which can fit inside the same couch recess without the coil holder after minor modifications of the couch overlay.

Despite the benefit of accommodating immobilization masks, replacing dedicated diagnostic head/neck coils with broadly applicable flexible coils may compromise image quality. Wong et al.^5^ have found reduced signal‐to‐noise ratio (SNR) and low‐contrast object detectability for images of the American College of Radiology (ACR) phantom obtained with a FL4 coil setup relative to a diagnostic head/neck coil at 1.5T. To select a flexible coil setup for MR simulation, it is important to compare image quality characteristics between the available coil options. Image SNR and contrast should be maximized for accurate tumor and organs at risk (OARs) detectability and delineation. On the other hand, setup feasibility, reproducibility, and patient comfort are additional criteria for successful clinical implementation of coils under consideration. While a higher number of receive coil elements is generally expected to provide better image quality, parameters such as coil geometry, filling factor, and coil quality factor also affect the results.^6^ Experimental evaluation using flexible coils shaped as intended for a particular MR examination is important for assessing clinically relevant image quality.

The goal of our study was to compare image quality between the commercial FL4 and the proposed novel UFL18 coil setup for brain and head and neck MR simulations at 3T, using a diagnostic head/neck coil as reference. Metrics were obtained using (1) the coil‐dependent parameters from a monthly ACR quality assurance (QA) procedure performed over 2 years, and (2) region of interest (ROI) assessments of SNR, contrast‐to‐noise ratio (CNR) and artifact size on images of five healthy volunteers, for clinically employed sequences. Image quality of the volunteer data was also reviewed by clinicians for treatment planning suitability.

## 2 MATERIALS AND METHODS

### 2.1 Coil setups

**FIGURE 1:**
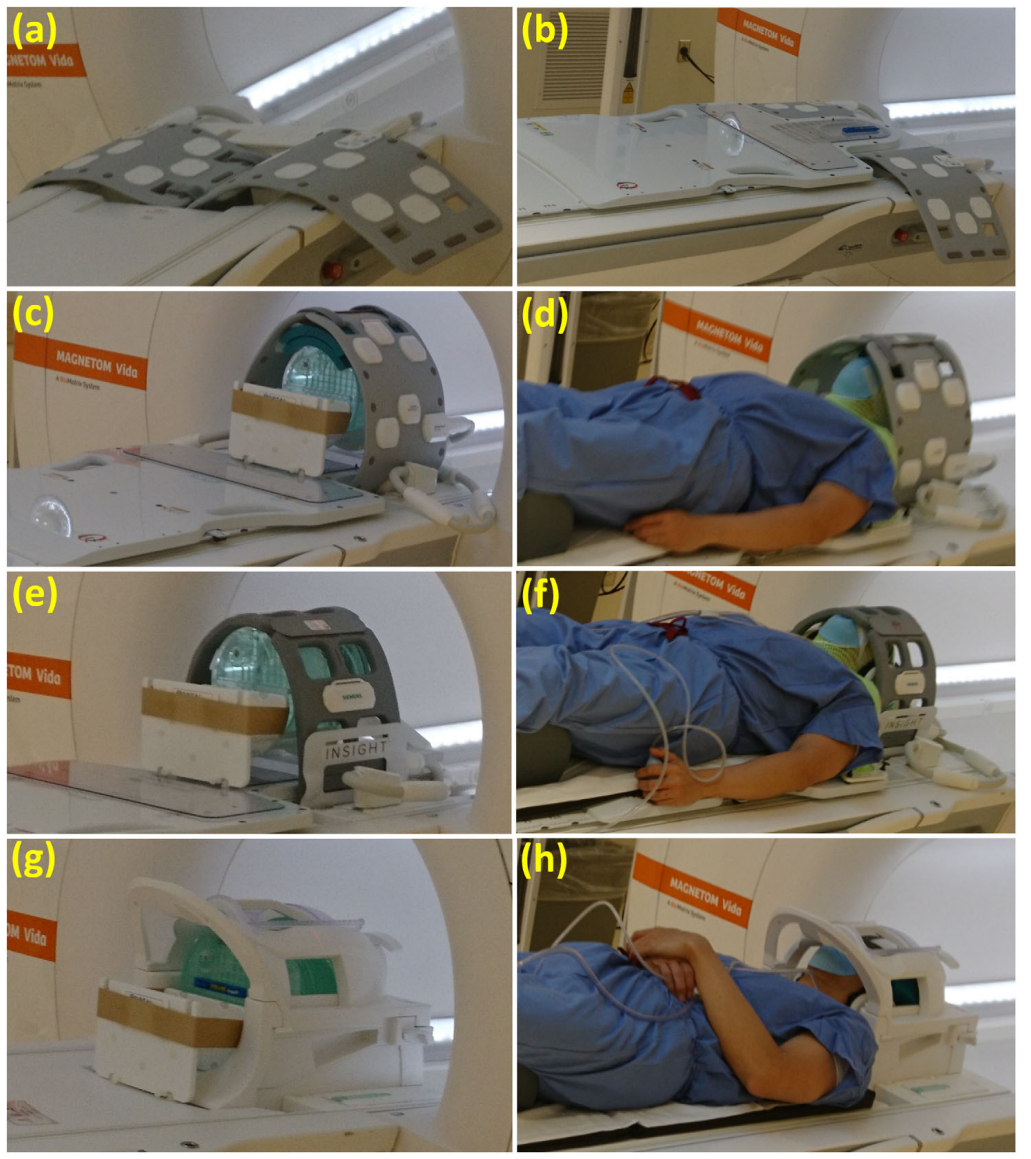
(a) Two UFL18 coils attached with Velcro straps side‐by‐side inside the head recess of a 3T Siemens Vida scanner couch. (b) The two coils under the INSIGHT overlay and Portrait MR Intracranial, Head & Neck Device used to support head/neck immobilization masks for radiotherapy simulations and treatment. (c and e) The UFL18 and FL4 coil ensemble, respectively, surrounding the large American College of Radiology (ACR) phantom in its cradle, placed on top of the immobilization boards. (d and f) A healthy volunteer in a thermoplastic mask for head imaging using the UFL18 and FL4 coil ensemble, respectively. (g) The ACR phantom in its cradle inside the HN20 coil, without immobilization boards. (h) The healthy volunteer as imaged by the HN20 coil without immobilization equipment

All imaging reported in this work was conducted in a 3T MAGNETOM Vida (Siemens Healthcare, Erlangen, Germany) with manufacturer provided coils. For the proposed UFL18 setup, two UltraFlex Large 18 coils of dimensions 290 mm × 590 mm each were attached side‐by‐side using Velcro straps and placed inside the head recess of the scanner couch, which has the same width (Figure 1a). An INSIGHT overlay (Qfix, Avondale, PA, USA) was modified by removing the two plastic pegs extending under each side of its head section, which are designed to fit into two slots on the head area of the scanner couch to fasten the board. Peg removal enabled overlay placement on top of the UFL18 coil ensemble. Velcro stickers were added on the underside of the overlay and on the surface of the scanner couch to warrant board stability. The Portrait MR Intracranial Head & Neck Device (Qfix, Avondale, PA, USA) on which thermoplastic masks are secured was then positioned on top of the overlay (Figure 1b). For the FL4 setup, the INSIGHT MR Coil Holder (Qfix, Avondale, PA, USA) was placed in the head recess of the scanner couch, and two Flex Large 4 coils (dimensions 224 mm × 516 mm each) were positioned side‐by‐side inside it, held together with Velcro straps. The modified INSIGHT overlay and Portrait board were then laid on top. The diagnostic setup consisted of a BioMatrix Head/Neck 20 (HN20) coil, placed inside the scanner couch recess without immobilization equipment.

### 2.2 Phantom study

Image quality for the three coil setups was assessed using the large MRI phantom for the ACR accreditation program, following the ACR recommended procedures.^7^ In the case of flexible coils, the phantom was positioned on top of the immobilization boards in a bespoke cradle, supported by a custom plexiglass block to achieve phantom horizontality. The phantom center markings were matched to the scanner positioning laser lines according to ACR recommendations and to the coil center markings. The two UFL18 coils were attached by Velcro straps above the phantom. Arched foam pads on the phantom top ensured that the coils were as equidistant from it as possible along their length (Figure 1c). The shorter FL4 coil setup was closed by a Velcro connecting piece (Figure 1e). The phantom in its cradle was placed inside the HN20 coil according to the standard ACR procedure, without immobilization boards (Figure 1g).

For each coil setup, the scanner couch was driven to the scanner isocenter. After a sagittal localizer, 11 axial slices of the T1‐weighted and T2‐weighted spin‐echo (SE) sequences recommended by ACR for the MRI accreditation program^7^ were acquired (Table 1). In addition, three sequences used clinically for treatment planning (a T1 and T2‐weighted turbo spin echo [TSE] and a short tau inversion recovery [STIR]) were slightly modified to obtain 11 axial slices matching the slice thickness, slice gap and field of view requirements for image quality assessments using the ACR phantom (table 1). The phantom was scanned successively with all three coil setups in the same day on roughly monthly intervals between 2019 and 2021, yielding 23 datasets of 5 image series for every coil.

**TABLE 1:** MR sequences employed in the present work and their parameters

Sequence	TR (ms)	TE (ms)	TI (ms)	FOV (mm^2^)	Matrix size	AF	ST (mm)	FA (°)	NA	BW (Hz/px)
ACR phantom										
ACR T1	500	20	–	250 × 250	256 × 256	–	5	90	1	150
ACR T2	2000	20	–	250 × 250	256 × 256	–	5	90	1	100
T1 TSE	616	9.3	–	249 × 249	352 × 352	2	5	135	2	237
T2 TSE	4280	109	–	250 × 250	288 × 288	2	5	120	2	200
STIR	4390	60	220	250 × 250	256 × 256	2	5	135	2	199
Healthy volunteers										
T1 TSE	669	9.7	–	240 × 240	352 × 352	2	4	135	2	237
T2 TSE	4970	108	–	239 × 239	288 × 288	2	4	120	2	200
STIR	3000	65	220	240 × 240	256 × 256	2	4	135	2	199

Abbreviations: ACR T1, ACR T2, T1‐weighted and T2‐weighted spin‐echo sequences recommended by the American College of Radiology MRI accreditation program; AF, acceleration factor; BW, bandwidth; FA, flip angle; FOV, field of view; NA, number of averages; STIR, short tau inversion recovery; TE, echo time; TI, inversion time; TR, repetition time; TSE, turbo spin echo; ST, slice thickness.

**FIGURE 2:**
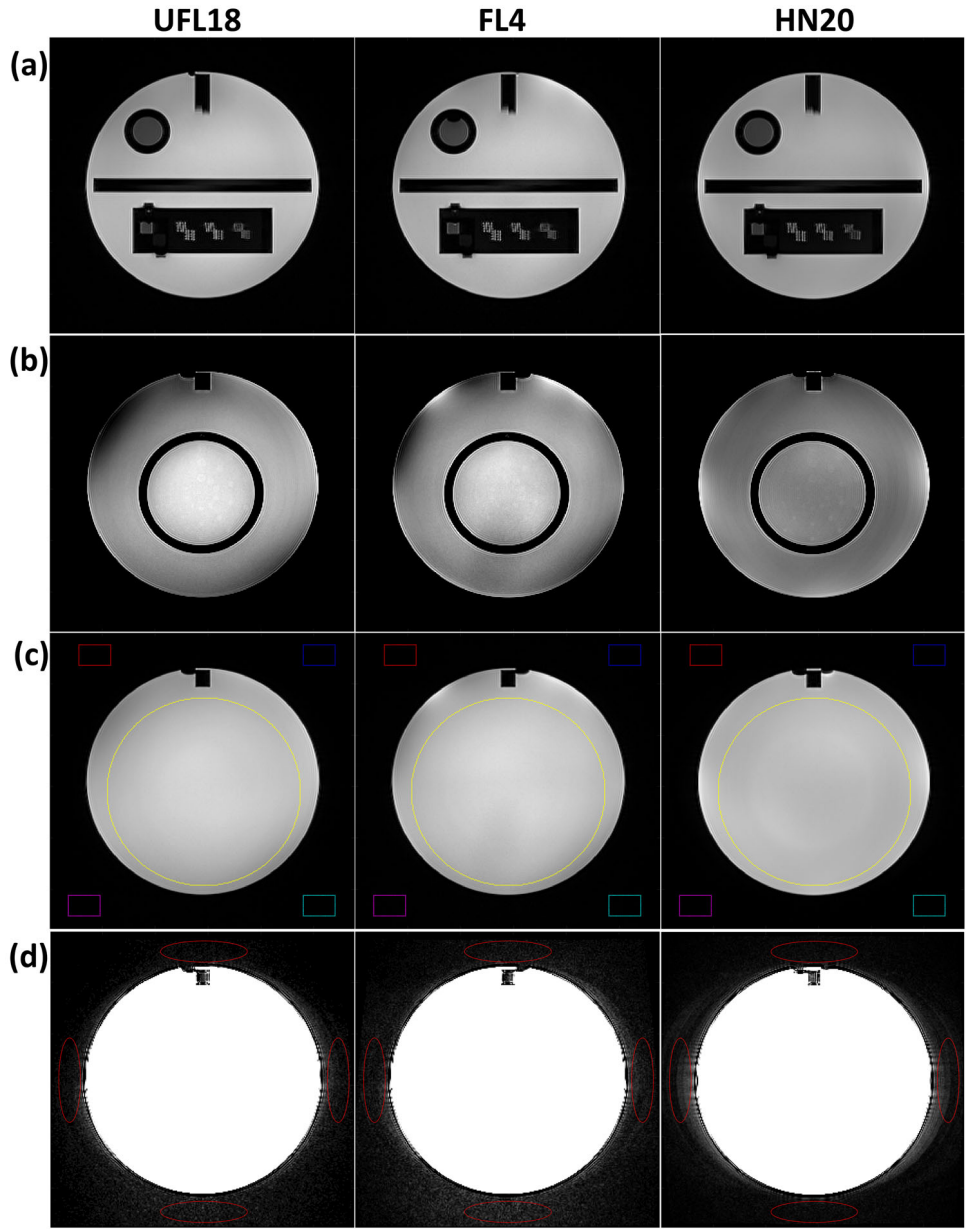
Example T2 turbo spin echo (TSE) images of the American College of Radiology (ACR) phantom, acquired with the UFL18 (left), FL4 (middle), and HN20 (right) coil setup. (a) Slice 1 employed for high‐contrast spatial resolution (HCSR) measurements, windowed in full dynamic range for each coil setup. (b) Slice 8 used for low‐contrast object detectability (LCOD) measurements, with displayed image intensities between 0.5 and 0.9 of the maximum of each image. (c) Slice 7 yielding uniformity and signal‐to‐noise ratio (SNR) calculations, individually windowed in full dynamic range for each coil setup. The yellow circular region of interest (ROI) provided the mean signal and the colorful rectangular ROIs a noise estimate for SNR. (d) Slice 7 windowed between 0 and 0.05 of the maximum signal intensity value for each image, with red elliptical ROIs demonstrating areas used for ghosting estimations.

All ACR phantom datasets were analyzed following the ACR accreditation program methodology.^7^ Analysis was performed in MATLAB R2020b (Natick, Massachusetts: The MathWorks Inc), using an adapted version of the OSAQA tool,^8^ whereas low‐contrast object detectability (LCOD) was assessed visually by the same observer. An additional SNR estimation was included for slice 7 of each sequence according to Clarke.^9^ Example phantom images obtained by the three coil setups for the same sequence are displayed in Figure 2, including regions of interest (ROIs) used for analysis. The ACR pass/fail criteria^7^ were applied on test results of the two ACR series for each coil setup.

Image quality between coil setups was compared for the 22 ACR phantom datasets remaining for each coil after discarding images of September 2020 when FL4 presented artifacts affecting background noise. The ACR phantom parameters, which may be affected by the receiving coil (high‐contrast spatial resolution [HCSR], image intensity uniformity [IIU], percent‐signal ghosting [PSG], LCOD and SNR), as well as geometric distortions, served as image quality criteria. Geometric distortions were calculated by subtracting the nominal 190 mm phantom diameter from each measured diameter^10^ on slices 1 and 5 of every acquired sequence according to the ACR geometric accuracy test.^7^ While IIU, PSG, LCOD, and SNR yielded one measurement per image series, HCSR yielded two (for the upper left and lower right hole array), and geometric accuracy yielded six (two diameters on slice 1 and four on slice 5 of the ACR phantom). For each parameter *p* and image series, the ratio of the parameter value obtained with the UFL18 or FL4 coil setup relative to its value for the HN20 coil (*p_UFL18_ / p_HN20_* or *p_FL4_ / p_HN20_*, respectively) was calculated. The parameter ratios of the two ACR sequences (44 for IIU, PSG, LCOD and SNR; 88 for HCSR; 264 for geometric distortions) and the parameter ratios of the three site sequences (66 for IIU, PSG, LCOD and SNR; 132 for HCSR; 396 for geometric distortions) were grouped together for each coil combination. Each *p_UFL18_ / p_HN20_* was compared against the corresponding *p_FL4_ / p_HN20_* for the ACR and clinical series using Wilcoxon rank‐sum tests with an alpha significance level of 0.05.

In addition, recorded specific absorption rate (SAR) values were extracted from the Digital Imaging and Communications in Medicine (DICOM) headers of each image series of the 22 ACR phantom datasets, providing a total of 110 datapoints for each coil setup. SAR had been calculated by the scanner software for the same dummy patient height and weight of 180 cm and 80 kg for all scans. Rank means of SAR between coil setups were statistically compared by Wilcoxon rank‐sum testing.

### 2.3 Volunteer study

Healthy volunteer scans were approved by the departmental Institutional Review Board (IRB) and five subjects (three males, two females) provided their informed consent. Each volunteer was imaged on the same day using three setups: treatment position (immobilization boards and thermoplastic mask) surrounded by the UFL18 or FL4 coil combination, and HN20 coil without treatment immobilization. Figure 1 shows an example volunteer in all three setups. When using the UFL18 combination, arched foam pads were placed on the volunteer's forehead to make the coil ensemble more symmetric, avoid direct skin contact with the coils, and create a feeling of a more spacious coil. The three clinical sequences employed in the phantom study (T1 and T2‐weighted TSE and STIR, table 1) were repeated with their original parameters for each coil setup, covering the head from the chin to the brain apex with two groups of 25 predominantly axial slices with no gaps. For two of the volunteers, the flip angle of the STIR sequence was automatically reduced in the FL4 case by the scanner Look Ahead SAR monitoring built‐in function; therefore the STIR data of these volunteers were discarded from further analysis.

**FIGURE 3:**
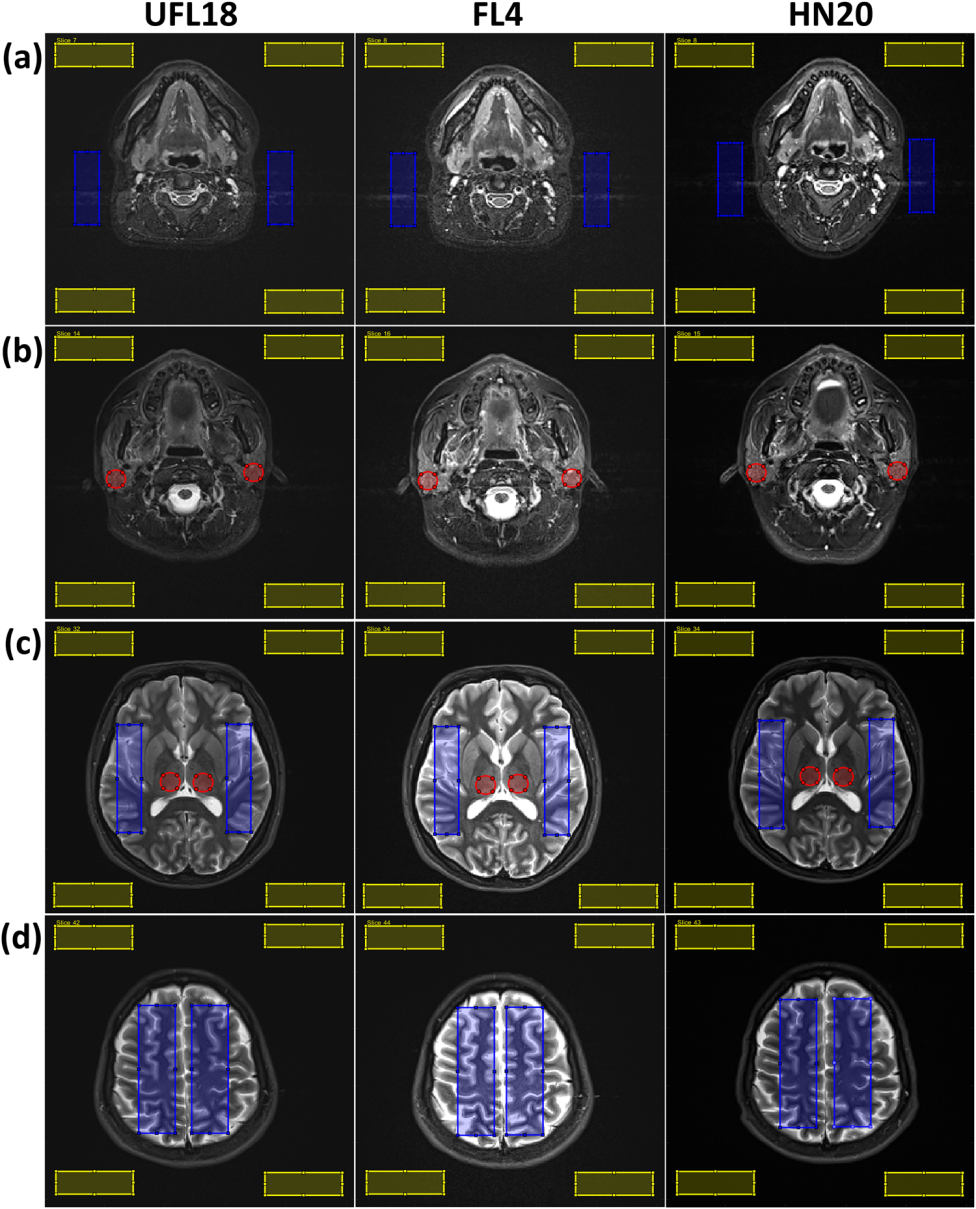
Axial short tau inversion recovery (STIR) images of a healthy volunteer for the three employed coil setups (left: UFL18; middle: FL4; right: HN20) with example regions of interest (ROIs) used for image quality assessments: (a) rectangular ROIs (blue) on areas affected by flow artifacts for artifact size calculations; (b) circular ROIs (red) on the parotids for signal‐to‐noise ratio (SNR) calculations. (c) Thalamus ROIs (red) used for SNR calculations, and brain ROIs (blue) employed for SNR and contrast‐to‐noise ratio (CNR) calculations, as also shown in (d). Yellow rectangular ROIs on the background provided noise estimations. The matching slices for UFL18 and FL4 had the same windowing, while the HN20 images were individually windowed.

Volunteer images of each sequence and coil setup were reviewed by a radiologist and a radiation oncologist for overall quality and treatment planning suitability, considering whether image signal and contrast were sufficient for distinguishing and delineating individual organs, and whether these organs were affected by artifacts. Quantitative image quality assessments were performed using custom MATLAB software. ROIs were defined for clinically relevant structures on single slices along the whole head: one circular ROI for the tongue base and brainstem; two circular ROIs for the right and left parotid, pterygoid muscle and thalamus; two rectangular ROIs for the right and left cerebellum; two rectangular ROIs for the right and left brain on four slices at different levels. Two rectangular ROIs were also placed on the background areas of three slices at different levels along the head, which presented flow or eye motion artifacts. The size of ROIs on a similar structure on both sides of the head was kept the same. Also, ROI size for a particular structure was equal between the matching slices of different sequences and coils for the same subject. Additional rectangular background ROIs were placed on the four corners of each slice selected for ROI evaluation, avoiding areas of signal truncation at image edges. Figure 3 demonstrates ROI examples for the same sequence and all coil setups.

Signal‐to‐noise ratio (SNR) for each tissue ROI was calculated by dividing its mean signal by the average standard deviation of the four background ROIs. The histogram spread of the cerebellum and brain ROIs served as contrast estimate^11^ for CNR calculations, using again the average standard deviation of the four background corner ROIs as a noise estimate. Histogram spread represented artifact size (AS) for the artifact ROIs. In total, 18 SNR, 10 CNR, and six artifact size measurements were obtained for each sequence of every subject and coil arrangement. Ratios of the SNR, CNR, and artifact size parameters for the UFL18 or FL4 arrangement relative to the parameters obtained with the HN20 coil for the same volunteer and sequence were formed. A total of 234 SNR ratios, 130 CNR ratios, and 78 artifact size ratios were obtained for each flexible coil ensemble over all sequences and volunteers. The parameter ratios for UFL18 were compared against those for FL4 using Wilcoxon rank‐sum tests (*α* = 0.05).

## 3 RESULTS

### 3.1 Phantom study

**FIGURE 4:**
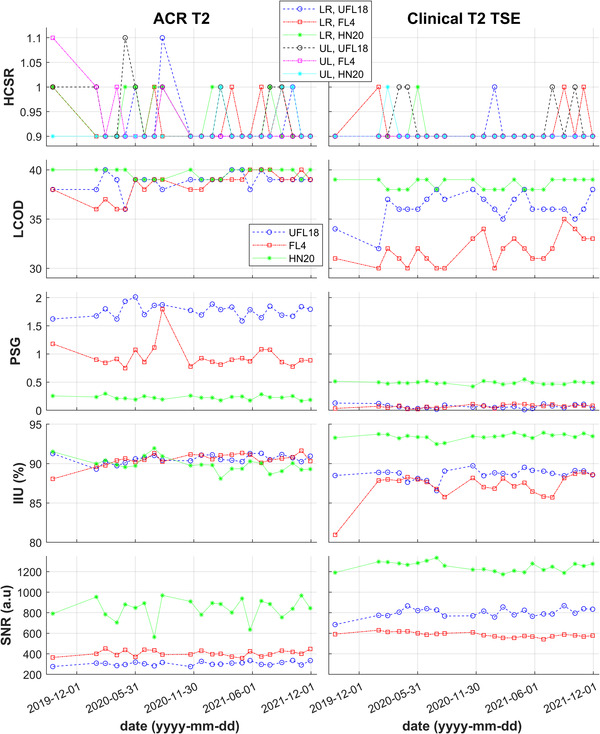
Graphs of high‐contrast spatial resolution (HCSR), low contrast object detectability (LCOD), percent‐signal ghosting (PSG), image intensity uniformity (IIU), and signal‐to‐noise ratio (SNR) over time, as measured with the American College of Radiology (ACR) phantom for the ACR T2‐weighted (left) and the clinical T2‐weighted (right) sequence. High‐contrast resolution is depicted with different colors for the lower right (LR) and the upper left (UL) array of the resolution insert. All other plots share the same legend, with UFL18, FL4, and HN20 indicated by blue circles, red squares, and green stars, respectively.

Table 2 lists the coil‐dependent and geometric accuracy parameters studied in this work, demonstrating that acceptance criteria were satisfied on average for all obtained parameters of the two ACR series (HCSR ≤ 1.0; LCOD ≥ 37; PSG ≤ 2.5; IIU ≥ 82.0%; 188 mm ≤ Diameter lengths ≤ 192 mm). Therefore all coil configurations have passed the ACR MRI Accreditation Program tests without needing to take the site series into account.^7^ Nevertheless, the clinical series demonstrated different parameter relations and opposite mean SNR between flexible coils than the ACR series, as also evident by the graphic comparison of coil‐dependent parameter values for the T2‐weighted sequences on Figure 4. Subtle differences in low‐contrast object detectability and image intensity uniformity between the two flexible coil setups for the ACR sequences became more pronounced for the clinical sequences, while SNR trends were reversed.

**TABLE 2:** Mean ± standard deviation of the coil dependent image quality parameters and of the diameter lengths for the geometric accuracy test, measured with the ACR phantom for the three coil setups, the two ACR sequences and three site sequences

**Parameter**	**Sequence**	**UFL18**	**FL4**	**HN20**
HCSR	ACR T1	0.94 ± 0.06	0.93 ± 0.05	0.91 ± 0.03
	ACR T2	0.93 ± 0.06	0.92 ± 0.05	0.93 ± 0.04
	T1 TSE	0.97 ± 0.08	0.95 ± 0.08	0.98 ± 0.08
	T2 TSE	0.91 ± 0.03	0.91 ± 0.03	0.90 ± 0.02
	STIR	0.91 ± 0.03	0.90 ± 0.02	0.90 ± 0.02
LCOD	ACR T1	39.4 ± 0.7	39.2 ± 1.0	40 ± 0.0
	ACR T2	38.9 ± 0.9	38.5 ± 1.3	39.6 ± 0.5
	T1 TSE	38.5 ± 1.0	36.9 ± 1.7	39.9 ± 0.3
	T2 TSE	36.2 ± 1.4	31.8 ± 1.5	38.5 ± 0.5
	STIR	32.8 ± 1.8	30.2 ± 1.7	37.9 ± 0.8
PSG	ACR T1	1.18 ± 0.10	0.63 ± 0.06	0.10 ± 0.03
	ACR T2	1.77 ± 0.11	0.96 ± 0.22	0.23 ± 0.03
	T1 TSE	0.03 ± 0.03	0.21 ± 0.04	0.03 ± 0.02
	T2 TSE	0.07 ± 0.03	0.07 ± 0.03	0.49 ± 0.03
	STIR	0.03 ± 0.02	0.14 ± 0.03	0.31 ± 0.02
IIU (%)	ACR T1	94.3 ± 0.4	93.9 ± 0.8	92.8 ± 0.7
	ACR T2	90.6 ± 0.5	90.5 ± 0.8	89.9 ± 0.9
	T1 TSE	86.1 ± 0.8	85.8 ± 1.5	93.0 ± 0.3
	T2 TSE	88.6 ± 0.7	87.2 ± 1.7	93.4 ± 0.4
	STIR	88.7 ± 0.7	87.2 ± 1.7	93.8 ± 0.3
SNR	ACR T1	501 ± 20	613 ± 28	1179 ± 69
	ACR T2	305 ± 18	406 ± 28	838 ± 105
	T1 TSE	1498 ± 100	1075 ± 44	2184 ± 60
	T2 TSE	802 ± 43	586 ± 23	1249 ± 45
	STIR	883 ± 79	571 ± 22	1168 ± 25
Diameter length	ACR T1	190.2 ± 0.8	190.2 ± 0.7	190.4 ± 0.7
	ACR T2	190.1 ± 0.8	190.1 ± 0.8	190.5 ± 0.7
	T1 TSE	190.4 ± 0.8	190.3 ± 0.7	190.7 ± 0.8
	T2 TSE	190.5 ± 0.8	190.5 ± 0.9	190.7 ± 0.7
	STIR	190.3 ± 0.8	190.3 ± 0.8	190.6 ± 0.7

Abbreviations: ACR, American College of Radiology; HCSR, High‐contrast spatial resolution; IIU, image intensity uniformity; LCOD, low‐contrast object detectability; PSG, percent‐signal ghosting; SNR, signal‐to‐noise ratio; STIR, short tau inversion recovery; TSE, turbo spin echo.

**FIGURE 5:**
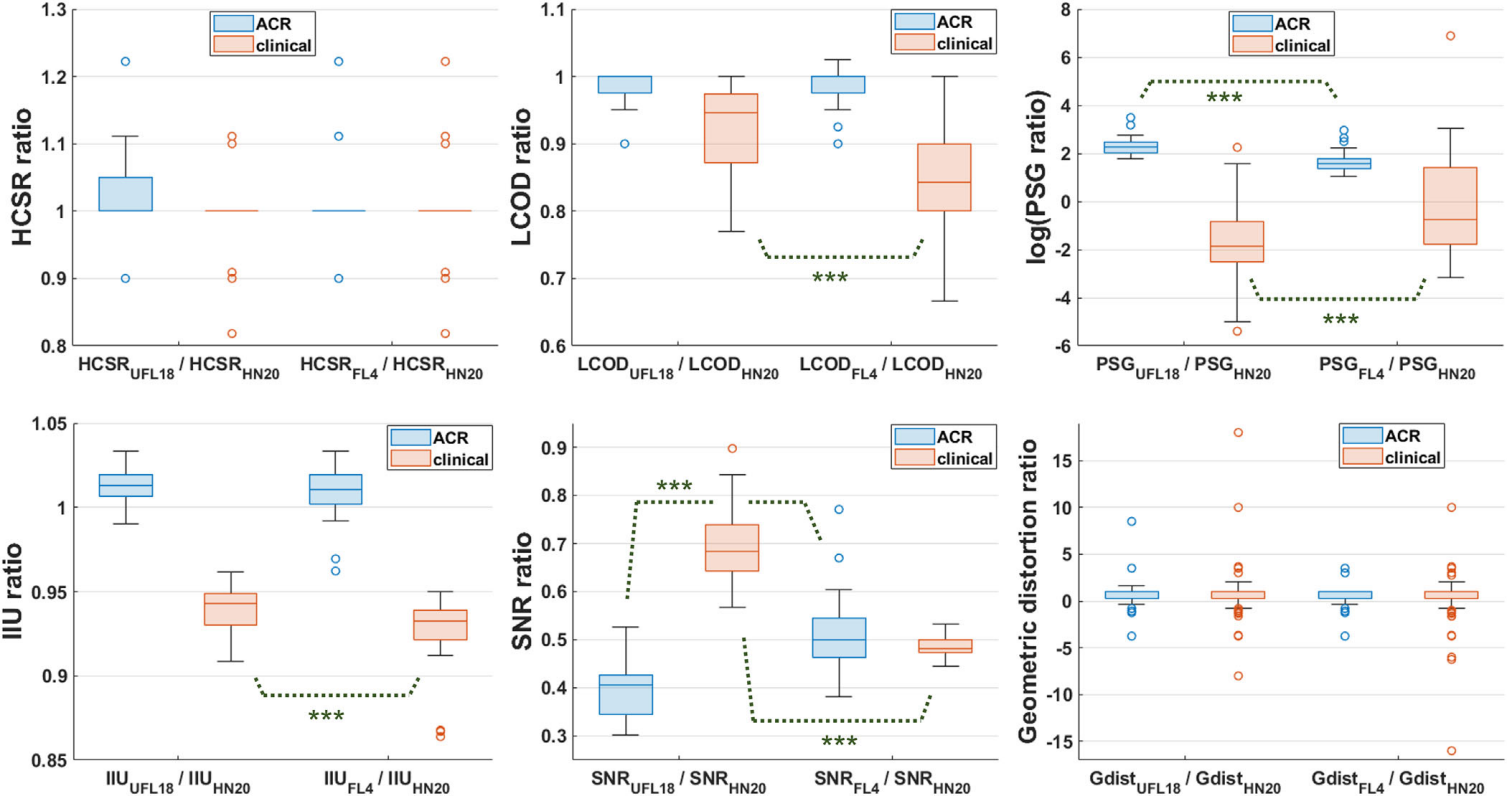
Boxplot charts for the ratios of high‐contrast spatial resolution (HCSR), low‐contrast object detectability (LCOD), percent‐signal ghosting (PSG) in semi‐logarithmic scale, image intensity uniformity (IIU), signal‐to‐noise ratio (SNR), and geometric distortion measured from American College of Radiology (ACR) phantom scans using the UFL18 or FL4 coil ensemble, over the corresponding values obtained by the HN20 coil. Blue and red boxes: parameter ratios obtained for the two ACR and the three clinical sequences, respectively; horizontal lines: medians; whiskers: minimum and maximum measurement; circles: outliers; ***statistically significant difference at *p* < 0.001 for the groups indicated by a dashed line

Boxplots summarizing the UFL18 and FL4 ratios of coil‐ dependent parameters and geometric distortions over their respective HN20 values are displayed in Figure 5 for the ACR and the clinical sequences. For high‐contrast spatial resolution, which has only 3 available ratings (0.9, 1.0, and 1.1), produced ratios could only vary between 0.818 and 1.222 and showed no statistically significant differences whether measured with the UFL18 or FL4 coil setup, for either ACR or clinical sequences (*z* = 0.97, *p* = 0.33, and *z* = 1.48, p = 0.14, respectively). Low‐contrast object detectability measured with UFL18 or FL4 yielded similar values to HN20 for the ACR sequences (LCOD_UFL18_ / LCOD_HN20_ = 0.98 ± 0.02; LCOD_FL4_ / LCOD_HN20_ = 0.98 ± 0.03), but its ratios were lower for the clinical sequences (LCOD_UFL18_ / LCOD_HN20_ = 0.92 ± 0.06; LCOD_FL4_ / LCOD_HN20_ = 0.85 ± 0.07). Wilcoxon rank‐sum tests indicated that LCOD_UFL18_ / LCOD_HN20_ was significantly higher than LCOD_FL4_ / LCOD_HN20_ (*z* = 5.48, *p* < 0.001) for the clinical sequences but not for the ACR sequences (*z* = 0.96, *p* = 0.34). Percent‐signal ghosting ratios presented large variations and outliers because PSG values measured with individual coil setups ranged in the order of 10^−4^ – 10^0^. On average, PSG_UFL18_ / PSG_HN20_ = 10.7 ± 5.1 and 0.65 ± 1.5; PSG_FL4_ / PSG_HN20_ = 5.7 ± 3.1 and 17.6 ± 120.5 for the ACR and clinical sequences, respectively. PSG_UFL18_ / PSG_HN20_ was significantly higher than PSG_FL4_ / PSG_HN20_ for the ACR sequences (*z* = 6.42, *p* < 0.001), but lower for the clinical sequences (*z* = −4.48, *p* < 0.001). Average image intensity uniformity was similar for all coils for the ACR sequences (both IIU_UFL18_ / IIU_HN20_ and IIU_FL4_ / IIU_HN20_ = 1.01 ± 0.01), and slightly lower with the flexible coils than with HN20 for the clinical sequences (IIU_UFL18_ / IIU_HN20_ = 0.94 ± 0.01; IIU_FL4_ / IIU_HN20_ = 0.93 ± 0.02). IIU ratios presented no statistically significant differences between flexible coil setups for the ACR sequences (*z* = 0.66, *p* = 0.51), while IIU_UFL18_ / IIU_HN20_ was higher than IIU_FL4_ / IIU_HN20_ for the site sequences (*z* = 4.33, *p* < 0.001). SNR was on average lower with the flexible coils than with HN20: SNR_UFL18_ / SNR_HN20_ = 0.40 ± 0.05 and 0.70 ± 0.07; SNR_FL4_ / SNR_HN20_ = 0.51 ± 0.07 and 0.48 ± 0.02 for the ACR and clinical sequences, respectively. Statistical tests showed that the SNR ratios for UFL18 were significantly lower than for FL4 for the ACR sequences (*z* = −6.50, *p* < 0.001), while the opposite was observed for the clinical sequences (*z* = 9.91, *p* < 0.001).

Geometric distortions, defined as the difference of measured from known phantom diameters, amounted on average to (0.2 ± 0.8) mm, (0.1 ± 0.7) mm, and (0.4 ± 0.7) mm for the ACR sequences acquired with UFL18, FL4 and HN20, respectively. For the clinical sequences, average geometric distortions with UFL18, FL4 and HN20 were (0.4 ± 0.8) mm, (0.4 ± 0.8) mm, and (0.6 ± 0.7) mm, respectively. No statistically significant differences were found between the ratios of geometric distortions obtained with the flexible coil setups over those obtained with the HN20 coil for neither ACR (*z* = −0.13, *p* = 0.89) nor clinical (*z* = 0.29, *p* = 0.77) sequences.

**FIGURE 6:**
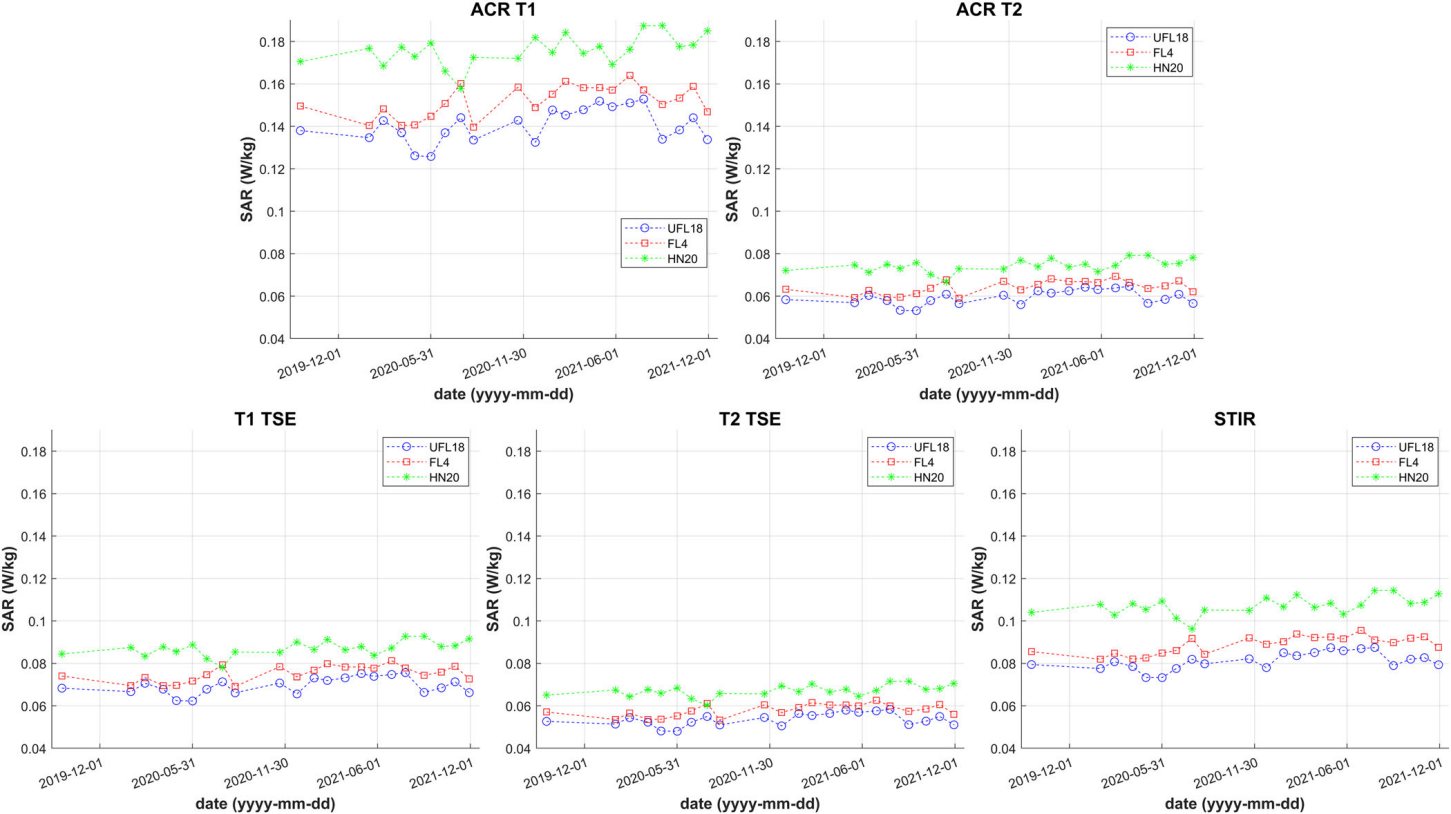
Plots of scanner calculated specific absorption rate (SAR) over all acquisitions of the American College of Radiology (ACR) T1 and T2 sequences and of the three site sequences with the ACR phantom for every coil setup. Datapoints corresponding to the UFL18, FL4, and HN20 coils are indicated by blue circles, red squares, and green stars, respectively.

SAR values calculated by the scanner software are displayed on Figure 6 for each sequence acquired using the ACR phantom and every coil setup. Average SAR for UFL18, FL4 and HN20 was (0.08 ± 0.03) W/kg, (0.09 ± 0.03) W/kg and (0.10 ± 0.04) W/kg, respectively. SAR with HN20 was significantly higher than with UFL18 (*z* = 5.69, *p* < 0.001) or FL4 (*z* = 4.14, *p* < 0.001), and FL4 SAR was significantly higher than UFL18 SAR (*z* = 2.55, *p* = 0.01).

### 3.2 Volunteer study

**FIGURE 7:**
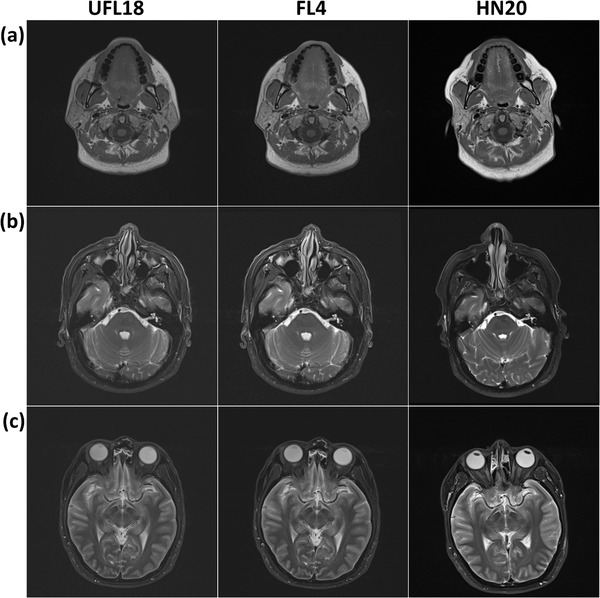
Example axial head images of a healthy volunteer acquired with the UFL18 (left), FL4 (middle), or HN20 (right) coil setup, using a T1 turbo spin echo (TSE) (a), T2 TSE (b), or short tau inversion recovery (STIR) (c) sequence. Slices were matched between coils using anatomical structures. Each image is displayed with its own default window width and level.

Figure 7 shows example images of the clinical sequences applied on a healthy volunteer. Head position was reproducible when using the flexible coil ensembles around immobilization equipment but varied for the diagnostic coil. Both reviewing clinicians found volunteer image quality acceptable for clinical practice.

**FIGURE 8:**
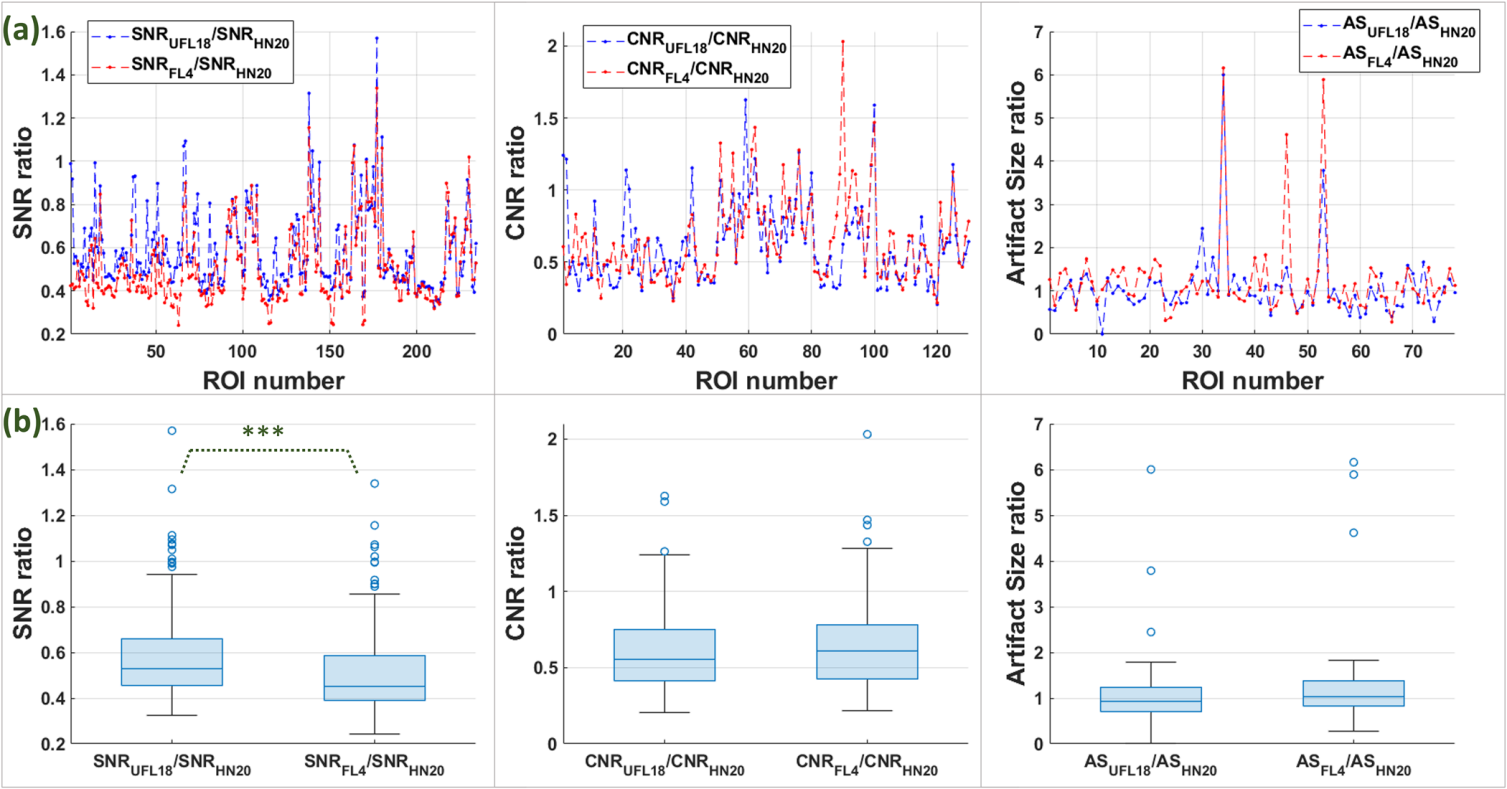
(a) Ratios of signal‐to‐noise ratio (SNR), contrast‐to‐noise ratio (CNR), and artifact size (AS) for UFL18 or FL4 (blue or red, respectively) relative to the corresponding values for HN20, over all regions of interest (ROIs) on healthy volunteer head images from which these parameters were extracted. (b) Boxplots of the same ratios. Blue lines: medians; whiskers: minimum and maximum measurement; blue circles: outliers; ***statistically significant difference at *p* < 0.001

Ratios of SNR, CNR, and artifact size obtained with the UFL18 or FL4 coil setup over those obtained with the HN20 coil are shown in Figure 8 over individual ROIs and in boxplot form. SNR obtained with the flexible coils was on average lower than with the dedicated HN20 coil: SNR_UFL18_ / SNR_HN20_ = 0.58 ± 0.19; SNR_FL4_ / SNR_HN20_ = 0.51 ± 0.18 (mean ± standard deviation). Wilcoxon rank‐sum tests revealed that SNR_UFL18_ / SNR_HN20_ was significantly larger than SNR_FL4_ / SNR_HN20_ (*z* = 5.50, *p* < 0.001). CNR was also lower with the flexible coil ensembles than with the standard head/neck coil (CNR_UFL18_ / CNR_HN20_ = 0.62 ± 0.28; CNR_FL4_ / CNR_HN20_ = 0.65 ± 0.29). Differences between the CNR ratios for UFL18 and FL4 were not statistically significant (*z* = −0.78, *p* = 0.44). Measured artifacts were slightly larger for the studied flexible coil setups than for HN20 (AS_UFL18_ / AS_HN20_ = 1.05 ± 0.8; AS_FL4_ / AS_HN20_ = 1.23 ± 0.9). No significant statistical differences were found between these artifact size ratios (*z* = −1.83, *p* = 0.07).

## 4 DISCUSSION

The large ACR MRI phantom was employed in this work as it is considered a standard test object for assessing and optimizing the quality of MR images obtained using head/neck coils. Its positioning mimicked the head position of human subjects by using the same immobilization boards and pads. Although a specific tabletop was modified to accommodate the custom UFL18 coil setup, other boards may be modified in a similar way to allow for flexible coils to surround them. The obtained ACR data are representative of long‐term coil function since they were acquired in regular intervals over 2 years. Data analysis was largely automated and thus more consistent and objective than manual measurements. Similarly to the findings of Wong et al.^5^ who have assessed a radiotherapy FL4 coil setup against a diagnostic HN20 coil at 1.5T, ACR criteria were satisfied for the ACR sequences at 3T for all coil configurations. Our study was more extensive, including clinical sequences, which yielded different results for the two studied flexible coil setups than the ACR sequences. While FL4 provided higher SNR ratios for the ACR series, UFL18 offered higher SNR ratios for the site series. Ghosting also presented opposite results for the different series, with UFL18 being overall less affected than FL4 for the clinical, but more for the ACR series. LCOD and uniformity ratios presented no statistical differences between the flexible coil setups for the ACR sequences but were higher for UFL18 for the clinical sequences.

The higher SNR observed with the FL4 setup for the unaccelerated ACR series may have been caused by the higher coil filling factor^6^ achieved with the shorter FL4 coils that surrounded the imaged object more closely. Differences in image quality characteristics between the flexible coil setups for the ACR and clinical series may be due to the acceleration employed in the clinical sequences, as the SNR of accelerated images benefits from increasing number of coil elements,^12^ and the UFL18 setup had a 4.5 times higher total number of receiving elements than the FL4 setup (36 against 8). Our results suggest that when using the ACR phantom for assessing the quality of MR images related to clinical practice, it is important to acquire not only the basic ACR recommended sequences but also the more complex sequences actually employed in the clinic.

Measured phantom diameters were similar for all coil setups, deviating on average by about half a millimeter or less from their nominal value. These small distortions imply that all employed coil setups provided sufficiently high image fidelity for radiotherapy treatment planning, which requires sub‐millimeter geometric accuracy within 10 cm from isocenter.^4^ The lack of statistically significant differences in geometric distortion ratios between the flexible coils agrees with expectations that distortions depend on factors unrelated to receiving coil selection (gradient nonlinearities, inhomogeneities of the static field B_0_, magnetic susceptibility of the imaged object, and acquisition bandwidth affecting chemical shift^4^, ^7^). Recorded SAR was overall higher for the diagnostic HN20 coil than for the two flexible coil setups, and higher for FL4 than for UFL18, despite using the same sequence and patient parameters for all ACR phantom scans. These differences may be explained by the fact that SAR estimation was affected by not only the positively rotating transmit field B1+ but also by the choice of receive coils.

Application of the clinical sequences on healthy volunteers revealed no significant differences of CNR and artifact size between flexible coil setups but corroborated the higher SNR ratios for UFL18 than FL4 observed on the phantom, despite small differences in sequence parameters. As the number of studied human subjects was small, statistical analysis was performed over all ROIs to increase sample size without differentiating between ROI positions. While this approach may not be informative for specific targets, it was sufficient for global image quality comparisons and coil setup selection. Areas of the neck below the chin were not assessed in this study, since they were not covered by either flexible coil setup. For clinical applications requiring neck imaging, additional flexible coils can be used on top of this body site.

The SNR observations for a T1 and T2‐weighted TSE and a STIR sequence in our work generally agreed with SNR comparisons between a similar UFL18 and a FL4 coil setup reported by Mengling et al.^13^ for a T1‐weighted MPRAGE and a T2‐weighted FLAIR sequence. Physicians found image quality suitable for treatment planning purposes in both studies, since lesions and organs at risk could be distinguished and contoured without being affected by artifacts. In our independent coil comparisons the UFL18 setup differed, because we modified the tabletop instead of designing a mask holder. Moreover, overlap of coil edges above a subject's face was avoided, as patients may find it more claustrophobic. We used foam pads on volunteers’ foreheads to increase distance from the coils and consequently minimize heating risks while making the UFL18 coil configuration more symmetric around the head and less confining. Unlike Mengling et al.,^13^ our data for all three coil configurations were acquired in the same day, allowing for more direct and accurate comparisons between coil setups. Our volunteer study was dedicated to coil assessments with the same sequences, and obtained images have not been affected by injected contrast agents as their patient images. We assessed not only SNR along the whole head, but also CNR for large ROIs containing gray and white matter, and artifacts unrelated to subject movement.

A direct comparison of obtained parameters between all coil setups was avoided because the diagnostic coil cannot be used with radiotherapy immobilization equipment. Considering HN20 as a gold standard, the performance of flexible coils was evaluated by forming image quality parameter ratios relative to it. Ratios were helpful for relating SNR and contrast metrics obtained from the phantom to those extracted from the volunteer data. ACR phantom and human subject results for clinical sequences showed that by using flexible coils, image SNR and CNR is about half, and ghosting is reduced relative to the diagnostic coil. Also, these coils provide about 90% of the uniformity and LCOD and similar artifact size as the diagnostic coil. This knowledge can be useful for deciding whether head position reproducibility with a flexible coil setup or higher SNR and CNR with the diagnostic coil is preferable when considering more demanding applications such as spectroscopy or diffusion‐weighted imaging.

Regarding choice of flexible coil setup, UFL18 provided overall better image quality parameters than FL4. Moreover, the novel UFL18 configuration was more spacious, accommodating volunteers with larger heads, and anecdotally perceived as less claustrophobic than the commercial FL4 solution. Since the UFL18 coils did not touch the volunteers’ skin, heating risks were reduced compared to the tighter FL4 coil setup. Additionally, on some instances of volunteer imaging, the scanner estimated SAR values for FL4 were higher than the corresponding limits, leading to flip angle reduction. These observations agree with the higher SAR recorded for FL4 than for UFL18 during the ACR phantom scans. Last but not least, UFL18 coil positioning was well reproducible between repeated phantom scans and for volunteers of different body sizes. After initial phantom and volunteer assessments the UFL18 coil setup has been successfully adopted in our clinical practice, while further phantom measurements were obtained for more comprehensive statistical comparisons.

## 5 CONCLUSION

A novel UFL18 coil setup provided overall better image quality than a commercial FL4 coil setup relative to a diagnostic HN20 coil for clinical sequences on the ACR phantom and healthy volunteers. The UFL18 coil arrangement was selected for clinical implementation in brain and head and neck MR simulations for radiotherapy treatment planning.

## AUTHOR CONTRIBUTIONS

Evangelia Kaza, Jeffrey Guenette, and Jonathan Schoenfeld contributed to the conception and design of the work and interpretation of the data. Evangelia Kaza acquired and analyzed the data and drafted the manuscript. Christian Guthier produced the MATLAB software for analyzing the volunteer data and contributed to interpretations of the results. Steven Hatch and Alexander Marques contributed to technical modifications of the UFL18 coil setup together with Evangelia Kaza and to acquisition of the volunteer data. Lisa Singer applied for the healthy volunteer IRB protocol and contributed to the conception of the work. In addition, all authors have revised the work critically, approved the final version and agree to be accountable for all aspects of the work.

## CONFLICT OF INTEREST

The authors declare no conflict of interest.
